# Supplementary material for: Integrated Identification and Genetic Diversity of Potentially Invasive Clearwing Moths (Lepidoptera: Cossoidea: Sesiidae) in Korea
Source: Insects. 2024 Jan 22;15(1):79. doi: 10.3390/insects15010079 (PMC10816279; doi:10.3390/insects15010079)
Supplement: Supplementary file 1 [file insects-15-00079-s001.zip › insects-2794857-supplementary.pdf]

Table S1. Information for all sequences used in molecular analyses.

No.	Sample ID	Scientific name	Genbank accession No. / BIN ID	Country
1	22SN1	*Sphecodoptera sheni*	PP003748 (this study)	South Korea
2	22SN2	*Sphecodoptera sheni*	PP003749 (this study)	South Korea
3	23BC3	*Sphecodoptera sheni*	PP003750 (this study)	South Korea
4	23BC4	*Sphecodoptera sheni*	PP003751 (this study)	South Korea
5	23BC5	*Sphecodoptera sheni*	PP003752 (this study)	South Korea
6	23BC6	*Sphecodoptera sheni*	PP003753 (this study)	South Korea
7	23BC7	*Sphecodoptera sheni*	PP003754 (this study)	South Korea
8	23BC8	*Sphecodoptera sheni*	PP003755 (this study)	South Korea
9	23CHC9	*Sphecodoptera sheni*	PP003756 (this study)	South Korea
10	23CHC10	*Sphecodoptera sheni*	PP003757 (this study)	South Korea
11	23CHC11	*Sphecodoptera sheni*	PP003758 (this study)	South Korea
12	23CHC12	*Sphecodoptera sheni*	PP003759 (this study)	South Korea
13	23CHC13	*Sphecodoptera sheni*	PP003760 (this study)	South Korea
14	23SN14	*Sphecodoptera sheni*	PP003761 (this study)	South Korea
15	23SN15	*Sphecodoptera sheni*	PP003762 (this study)	South Korea
16	23SN16	*Sphecodoptera sheni*	PP003763 (this study)	South Korea
17	23SN17	*Sphecodoptera sheni*	PP003764 (this study)	South Korea
18	23SN18	*Sphecodoptera sheni*	PP003765 (this study)	South Korea
19	23SN19	*Sphecodoptera sheni*	PP003766 (this study)	South Korea
20	23SN20	*Sphecodoptera sheni*	PP003767 (this study)	South Korea
21	CJ1S	*Sphecodoptera sheni*	PP003768 (this study)	South Korea
22	CJ2S	*Sphecodoptera sheni*	PP003769 (this study)	South Korea
23	DG1S	*Sphecodoptera sheni*	PP003770 (this study)	South Korea
24	DG2S	*Sphecodoptera sheni*	PP003771 (this study)	South Korea
25	DG3S	*Sphecodoptera sheni*	PP003772 (this study)	South Korea
26	DG4S	*Sphecodoptera sheni*	PP003773 (this study)	South Korea
27	DG5S	*Sphecodoptera sheni*	PP003774 (this study)	South Korea
28	DG6S	*Sphecodoptera sheni*	PP003775 (this study)	South Korea
29	IPE4W4S	*Sphecodoptera sheni*	PP003776 (this study)	South Korea
30	IPE5W5S	*Sphecodoptera sheni*	PP003777 (this study)	South Korea
31	IPE6W6S	*Sphecodoptera sheni*	PP003778 (this study)	South Korea
32	Ilsa7S	*Sphecodoptera sheni*	PP003779 (this study)	South Korea
33	Ilsan8S	*Sphecodoptera sheni*	PP003780 (this study)	South Korea
34	Ilsan9S	*Sphecodoptera sheni*	PP003781 (this study)	South Korea
35	Ilsan10S	*Sphecodoptera sheni*	PP003782 (this study)	South Korea
36	Ilsan11S	*Sphecodoptera sheni*	PP003783 (this study)	South Korea
37	BWLW7S	*Sphecodoptera sheni*	PP003784 (this study)	South Korea
38	BWLW8	*Sphecodoptera sheni*	PP003785 (this study)	South Korea

39	IPE3W3P	*Paranthrenella* sp. n.	PP003786 (this study)	South Korea
40	BWLW9P	*Paranthrenella* sp. n.	PP003787 (this study)	South Korea
41	BWLW10P	*Paranthrenella* sp. n.	PP003788 (this study)	South Korea
42	JH3Aj	*Anatrachyntis japonica*	PP003789 (this study)	South Korea
43	JH4Aj	*Anatrachyntis japonica*	PP003790 (this study)	South Korea
44	GSCMB812-12	*Sphecodoptera sheni*	BOLD:ABW9347	China
45	ANICF612-10	*Paranthrenella chrysophanes*	HQ922206/ BOLD:AAI7641	Australia
46	ANICF613-10	*Paranthrenella chrysophanes*	HQ922207/ BOLD:AAI7641	Australia
47	ANICF615-10	*Paranthrenella chrysophanes*	HQ922208/ BOLD:AAI7641	Australia
48	LTOLB087-08	*Paranthrenella chrysophanes*	KF522587/ BOLD:AAI7641	Australia
49	EPNG8497-14	*Paranthrenella auriplena*	BOLD:ACO5543	Papua New Guinea
50	GBMNE5028-21	*Paranthrenella cinnamoma*	MW315912/ BOLD:AEN4976	China
51	GBMNE61298-22	*Paranthrenella formosicola*	OM401537/ BOLD:AEY6232	Taiwan
52	EPNG8498-14	*Paranthrenella terminalia*	BOLD:ACO5542	Papua New Guinea

Table S2. Haplotype distribution in this study.

Haplotype_number	No. of sequences	Sample ID (for each individual)
Hap_1:	30	22SN1, 22SN2, 23BC3, 23BC4, 23BC5, 23BC6, 23BC8, 23CHC9, 23CHC11, 23CHC12, 23CHC13, 23SN14, 23SN15, 23SN16, 23SN17, 23SN18, 23SN19 23SN20 CJ2S DG1S DG2S DG3S DG4S DG5S DG6S IPE4W4S IPE5W5S Ilsan8S Ilsan9S Ilsan10S
Hap_2:	2	23BC7, CJ1S
Hap_3:	1	23CHC10
Hap_4:	1	IPE6W6S
Hap_5:	1	Ilsa7S
Hap_6:	1	Ilsan11S
Hap_7:	2	BWLW7S, BWLW8S
Hap_8:	1	GSCMB812-12
Hap_9:	1	IPE3W3P
Hap_10:	2	BWLW9P, BWLW10P
Hap_11:	2	ANICF612-10, ANICF615-10
Hap_12:	1	ANICF613-10
Hap_13:	1	LTOLB087-08
Hap_14:	1	EPNG8497-14
Hap_15:	1	GBMNE5028-21
Hap_16:	1	EPNG8498-14
Hap_17:	1	GBMNE61298-22
Hap_18:	2	JH3Aj, JH4Aj
